# Association between IL4, IL6 gene polymorphism and lumbar disc degeneration in Chinese population

**DOI:** 10.18632/oncotarget.21650

**Published:** 2017-10-06

**Authors:** Yong Zhu, Shunan Li, FangLin Niu, Mengdan Yan, Jing Li, Haiyu Jia, Xuejun Yang

**Affiliations:** ^1^ The Second Affiliated Hospital of Inner Mongolia Medical University, Hohhot 010030, China; ^2^ The Hohhot First Hospital, Hohhot 010020, China; ^3^ Key Laboratory of Resource Biology and Biotechnology in Western China (Northwest University), Ministry of Education, Xi’an 710069, China; ^4^ The Affiliated Hospital of Inner Mongolia Medical University, Hohhot 010020, China

**Keywords:** lumbar disc disease (LDD), IL4, IL6, Han Chinese, case-control

## Abstract

Lumbar disc disease (LDD) is a common musculoskeletal disorder, caused by degeneration of intervertebral discs of the lumbar spine and is one of the most common musculoskeletal disorders affliction in adult. There is growing evidence that LDD has strong genetic determinants. We analyze whether the IL4 and IL6 gene polymorphism is related to LDD in Chinese Han population. The participants were 498 with LDD and 463 without LDD. IL4 and IL6 gene polymorphism were determined by Sequenom MassARRAY. We found that SNPs rs1800796(OR = 1.29, 95% CI, 1.07 – 1.57, p = 0.009), rs1524107(OR = 1.28, 95% CI, 1.05 – 1.55, p = 0.013), rs2069840 (OR = 1.39, 95% CI, 1.03 – 1.89, p = 0.033) in IL6 gene were significantly associated with LDD risk at a 5% level. In addition, genetic models found IL4 gene (rs2243250) were associated with LDD. In this study, we analyzed and associated SNPs of IL4 and IL6 with LDD risk. In summary, four variations (rs1800796, rs1524107, rs2069840, rs2243250) of the selected candidate SNPs were associated with susceptibility to LDD in our study. The results of this study have the guiding significance in clinical work in the future in the treatment of lumbar disc herniation patients, not one-sided that the symptoms of low back pain only from mechanical oppression.

## INTRODUCTION

Lumbar disc disease (LDD) is a common musculoskeletal disorder, caused by degeneration of intervertebral discs of the lumbar spine and is one of the most common musculoskeletal disorders affliction in adults [1, 2]. Etiology of degenerative disc disease is complex, it has been suggested that some environmental factors, such as physical loading [3], obesity [4], smoking [5] et al. may play a role. Although various environmental have been implicated in the pathogenesis of disc degeneration, there is growing evidence that LDD has strong genetic determinants [6].

A great many genes have now been shown to be related to lumbar disc herniation, including MMP [7, 8], VDR [9], FAS [10] and COL [11] et al. It is generally believed that neuroin- flammation plays a crucial role in the development of chronic pain [12]. It has been more and more is convinced of that lumbar disc pain may be pathophysiologically induced by chemical inflammation, inflammatory cytokines are not only closely related to other cytokines and chemical mediators but also play an important role in lumbar disc herniation, although the inflammatory factors that contribute to lumbar disc herniation and pain remain to be determined [13]. Anthi Kelempisioti et al. [14] reported the associations of IL6 genes for DD among young adults in Northern Finland and indicated that IL6 is involved in the etiology of DD among young adults. Pasi J Eskola et al.[15] paper was to examine the associations between IL6 polymorphisms and LDD, the results suggest possible roles for IL6 in early DD among girls. Burke et al.[16]showed that herniated disc cells secrete large amounts of IL6 and other inflammatory mediators and cytokines that cause lumbar pain. Kang et al.[17] demonstrated that IL-6 was expressed in both normal and herniated discs, but was significantly induced in the herniated discs. Kraychete et al. [18]showed that patients with chronic low back pain due to disc herniation had high levels of TNF-α and IL-6.Thus, this study assessed single-nucleotide polymorphisms (SNPs) in the IL-4 and IL-6 with LDD risk in a Chinese Han population. It is well investigated that cytokines play a role in LDD.

## RESULTS

Table 1 give the volunteers characteristics, four hundred and ninety-eight patients with LDD (200 females, 298 males, 50.27±12.53) and four hundred and sixty-three gender-matched(P=0.978) and age(P=0.413) and healthy adults (198 females, 265 males, 50.65±11.79), were enrolled in this case-control study. The allele frequencies and characteristics of IL4 and IL6 SNPs are shown in Table 2. The genotype frequencies fit in with the Hardy–Weinberg equilibrium. Using the χ2 test, three SNPs rs1800796(OR = 1.29, 95% CI, 1.07 – 1.57, p = 0.009), rs1524107(OR = 1.28, 95% CI, 1.05 – 1.55, p = 0.013), rs2069840 (OR = 1.39, 95% CI, 1.03 – 1.89, p = 0.033) in IL6 gene were significantly associated with LDD risk at a 5% level.

**Table 1:** Characteristics of LDD patients and control participants

	Case	Control	P
	498	463	
Gender			0.413
Male	298	265	
Female	200	198	
Age	50.27±12.53	50.65±11.79	0.978

p ≤ 0.05 indicates statistical significance.

p was calculated by Pearson’s χ2 test.

**Table 2:** Basic information on candidate SNPs and their association with LDD risk in this study

SNP	Gene	Chr	Allele	MAF(case)	MAF(control)	HWE	OR	95%CI	P
rs2243250	IL4	5q31.1	C/T	0.211	0.245	0.131	0.82	0.67-1.02	0.073
rs2227284	IL4	5q31.1	G/T	0.16	0.172	0.100	0.92	0.72-1.17	0.477
rs2243267	IL4	5q31.1	G/C	0.212	0.240	0.253	0.85	0.69-1.06	0.144
rs2243270	IL4	5q31.1	A/G	0.212	0.240	0.253	0.85	0.69-1.06	0.144
rs2243283	IL4	5q31.1	G/C	0.161	0.185	0.120	0.84	0.66-1.07	0.156
rs2243289	IL4	5q31.1	A/G	0.211	0.234	0.244	0.87	0.70-1.08	0.216
rs1800796	IL6	7p15.3	G/C	0.344	0.288	0.309	1.29	1.07-1.57	0.009^*^
rs2069837	IL6	7p15.3	G/A	0.203	0.189	0.097	1.09	0.87-1.37	0.452
rs1524107	IL6	7p15.3	T/C	0.35	0.297	0.025	1.28	1.05-1.55	0.013^*^
rs2069840	IL6	7p15.3	G/C	0.112	0.083	0.116	1.39	1.03-1.89	0.033^*^

MAF: minor allele frequency; OR: odds ratio; 95% CI: 95% confidence interval. * p < 0.05 indicates statistical significance.

HWE p ≤ 0.01 is excluded.

Next, we assumed that the minor allele of each SNP was a risk factor and four genetic models (co-dominant, dominant, recessive and additive) were used to further identify the associations between the SNPs and the LDD risk in Table 3. Four susceptibility SNPs were associated with LDD risk. For rs2243250 in IL4, the genotype “CC” compared with “TT/CT” was associated with a decreased risk of LDD in the recessive model before and after adjustment (OR = 0.50, 95% CI, 0.28 – 0.89, p = 0.016). In IL6 gene, we found three SNPs were increased the LDD risk. For rs1800796, the individual carraying “C” allele compared with “G” allele increased the LDD risk (Log-additive, OR =1.29, 95% CI, 1.07 – 1.57, p = 0.0097) by unconditional logistic regression adjusted for age and gender. The “C” allele of rs1524107 and the “G” allele of rs2069840 showed significantly increased risk of LDD (OR= 1.26, 95%CI = 1.04 - 1.53, P= 0.016; OR= 1.39, 95%CI: 1.02-1.89, P= 0.034, respectively).

**Table 3:** Association between IL4, IL6 genotypes and LDD risk under different genotypic models

	Model	Genotype	Control	Case	OR^a^ (95% CI)	P^a^-value	OR^b^ (95% CI)	P^b^-value
rs2243250	Codominant	T/T	270 (58.3%)	307 (61.6%)	1	0.052	1	0.052
		C/T	159 (34.3%)	172 (34.5%)	0.95 (0.73-1.25)		0.95 (0.72-1.24)	
		C/C	34 (7.3%)	19 (3.8%)	0.49 (0.27-0.88)		0.49 (0.27-0.88)	
	Dominant	T/T	270 (58.3%)	307 (61.6%)	1	0.29	1	0.28
		C/T-C/C	193 (41.7%)	191 (38.4%)	0.87 (0.67-1.13)		0.87 (0.67-1.12)	
	Recessive	T/T-C/T	429 (92.7%)	479 (96.2%)	1	0.016	1	0.016
		C/C	34 (7.3%)	19 (3.8%)	0.50 (0.28-0.89)		0.50 (0.28-0.89)	
	Log-additive	---	---	---	0.83 (0.67-1.02)	0.076	0.82 (0.67-1.02)	0.073
rs1800796	Codominant	C/C	239 (51.6%)	210 (42.2%)	1	0.015	1	0.016
		G/C	181 (39.1%)	232 (46.7%)	1.46 (1.12-1.91)		1.45 (1.11-1.90)	
		G/G	43 (9.3%)	55 (11.1%)	1.46 (0.94-2.26)		1.45 (0.93-2.26)	
	Dominant	C/C	239 (51.6%)	210 (42.2%)	1	0.0036	1	0.004
		G/C-G/G	224 (48.4%)	287 (57.8%)	1.46 (1.13-1.88)		1.45 (1.13-1.88)	
	Recessive	C/C-G/C	420 (90.7%)	442 (88.9%)	1	0.36	1	0.37
		G/G	43 (9.3%)	55 (11.1%)	1.22 (0.80-1.85)		1.21 (0.79-1.85)	
	Log-additive	---	---	---	1.29 (1.07-1.57)	0.0088	1.29 (1.06-1.57)	0.0097
rs1524107	Codominant	T/T	238 (51.6%)	207 (41.6%)	1	0.0055	1	0.0063
		C/T	172 (37.3%)	233 (46.8%)	1.56 (1.19-2.04)		1.55 (1.18-2.03)	
		C/C	51 (11.1%)	58 (11.7%)	1.31 (0.86-1.99)		1.30 (0.85-1.98)	
	Dominant	T/T	238 (51.6%)	207 (41.6%)	1	0.0018	1	0.0021
		C/T-C/C	223 (48.4%)	291 (58.4%)	1.50 (1.16-1.94)		1.49 (1.16-1.93)	
	Recessive	T/T-C/T	410 (88.9%)	440 (88.3%)	1	0.78	1	0.78
		C/C	51 (11.1%)	58 (11.7%)	1.06 (0.71-1.58)		1.06 (0.71-1.58)	
	Log-additive	---	---	---	1.27 (1.05-1.53)	0.014	1.26 (1.04-1.53)	0.016
rs2069840	Codominant	C/C	391 (84.6%)	386 (78.1%)	1	0.0087	1	0.0095
		G/C	65 (14.1%)	105 (21.3%)	1.64 (1.16-2.30)		1.63 (1.16-2.29)	
		G/G	6 (1.3%)	3 (0.6%)	0.51 (0.13-2.04)		0.51 (0.13-2.04)	
	Dominant	C/C	391 (84.6%)	386 (78.1%)	1	0.0098	1	0.011
		G/C-G/G	71 (15.4%)	108 (21.9%)	1.54 (1.11-2.15)		1.53 (1.10-2.14)	
	Recessive	C/C-G/C	456 (98.7%)	491 (99.4%)	1	0.27	1	0.27
		G/G	6 (1.3%)	3 (0.6%)	0.46 (0.12-1.87)		0.46 (0.12-1.87)	
	Log-additive	---	---	---	1.39 (1.03-1.90)	0.032	1.39 (1.02-1.89)	0.034

OR: odds ratio; 95% CI: 95% confidence interval. * *p* < 0.05 indicates statistical significance.

^a^: calculated from two-sided chi-square tests or Fisher's exact tests for either genotype distribution.

^*b*^: calculated by unconditional logistic regression adjusted for age and sex.

**Figure 1:**
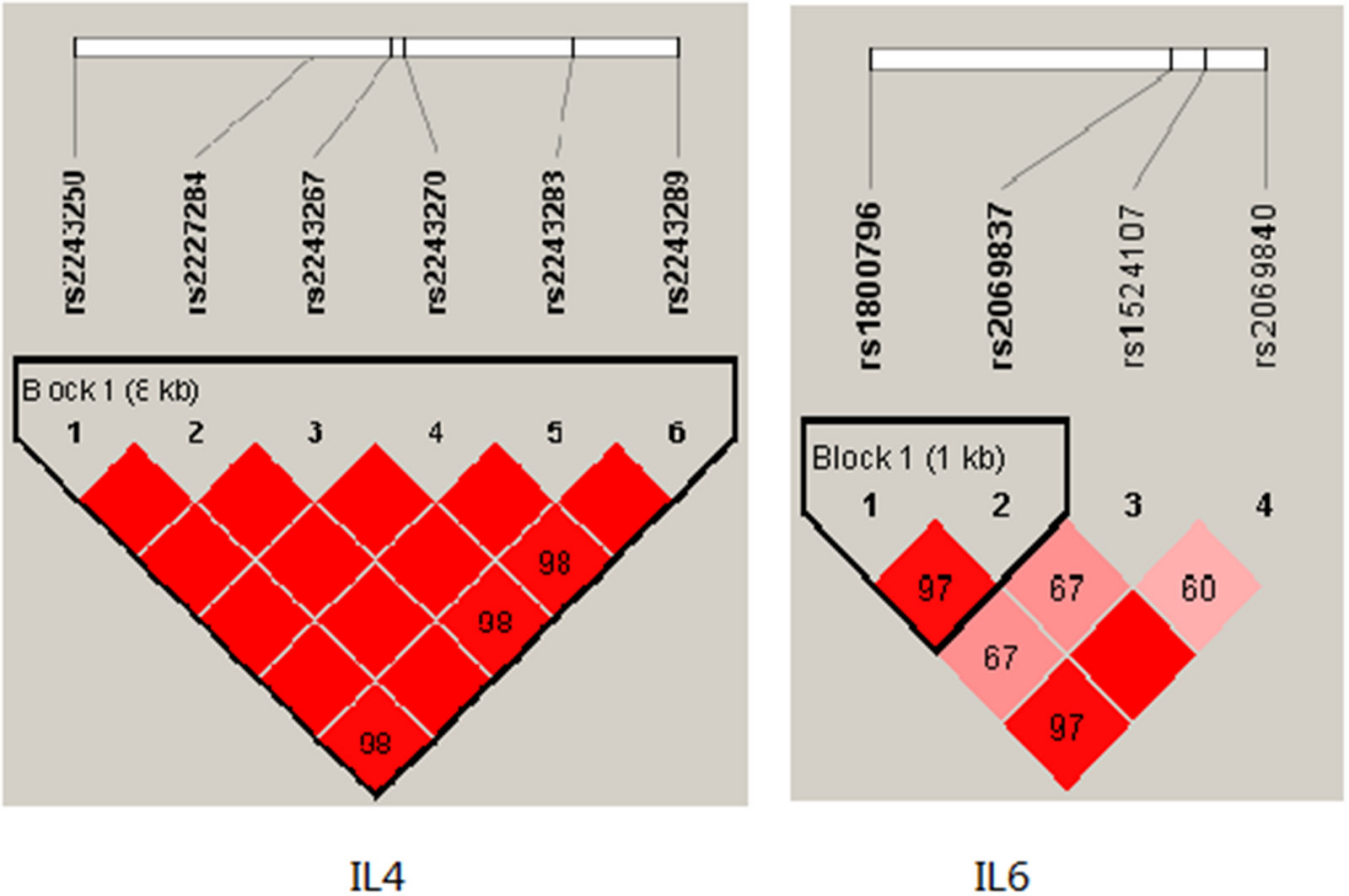
Haplotype block map for the IL4, IL6 SNPs genotyped in this study

Furthermore, Linkage disequilibrium (LD) and haplotype analyses of the SNPs were further studied. The LD block in Figure 1. In Table 4 haplotype analyses of the SNPs, we found the “CA” haplotype of rs1800796, rs2069837 was associated with LDD risk before and after the adjustment (adjusted OR= 1.56, 95%CI: 1.17-2.08, *P*= 0.0026).

**Table 4:** *IL6* haplotype frequencies and their associations with LDD risk

	rs1800796	rs2069837	Freq	OR^a^ (95% CI)	P^a^-value	OR^b^(95% CI)	P^b^-value
1	C	A	0.6787	1	---	1	---
2	G	G	0.192	1.16 (0.93 - 1.46)	0.19	1.16 (0.93 - 1.46)	0.190
3	G	A	0.1252	1.57 (1.18 - 2.09)	0.0022	1.56 (1.17 - 2.08)	0.003

OR: odds ratio; 95% CI: 95% confidence interval.

^a^: calculated from two-sided chi-square tests or Fisher’s exact tests for either genotype distribution.

^*b*^: calculated by unconditional logistic regression adjusted for age and sex.

Finally, we analyzed the effect of these SNPs and LDD association by age and gender stratification in Table 5. We found only one SNP (rs2069840, IL6, P = 0.019) polymorphism were associated with LDD in age < 50 population, meanwhile for age > 50 two SNPs were found siginification (rs1800796, P = 0.029; rs1524107, P = 0.020). For female we did not found any SNPs were associated with LDD, however for male population three SNPs were found (rs1800796, P= 0.018; rs1524107, P=0.040; rs2069840, P=0.045).

**Table 5:** The association between SNPs and age, gender analysis of LDD patients

SNP	Gene	Allele	≤50			>50			Male			Female		
			OR	95%CI	P	OR	95%CI	P	OR	95%CI	P	OR	95%CI	P
rs2243250	IL4	C/T	0.88	0.65-1.20	0.430	0.77	0.57-1.04	0.084	0.87	0.66-1.15	0.320	0.76	0.55-1.06	0.110
rs2227284	IL4	G/T	1.05	0.74-1.49	0.776	0.81	0.58-1.13	0.208	1.01	0.74-1.37	0.968	0.78	0.53-1.15	0.215
rs2243267	IL4	G/C	0.89	0.66-1.22	0.479	0.81	0.61-1.10	0.177	0.92	0.7-1.22	0.575	0.76	0.55-1.06	0.109
rs2243270	IL4	A/G	0.89	0.66-1.22	0.479	0.81	0.61-1.10	0.177	0.92	0.7-1.22	0.575	0.76	0.55-1.06	0.109
rs2243283	IL4	G/C	0.81	0.57-1.15	0.243	0.87	0.63-1.20	0.394	0.76	0.56-1.04	0.085	0.97	0.67-1.41	0.872
rs2243289	IL4	A/G	0.94	0.69-1.28	0.701	0.81	0.60-1.10	0.177	0.93	0.70-1.24	0.628	0.79	0.57-1.11	0.174
rs1800796	IL6	G/C	1.25	0.93-1.66	0.134	1.34	1.03-1.74	0.029	1.35	1.05-1.73	0.018	1.20	0.88-1.63	0.246
rs2069837	IL6	G/A	0.89	0.63-1.26	0.524	1.27	0.94-1.71	0.123	1.05	0.79-1.41	0.726	1.14	0.80-1.63	0.459
rs1524107	IL6	T/C	1.18	0.89-1.57	0.257	1.36	1.05-1.77	0.020	1.30	1.01-1.66	0.040	1.23	0.91-1.67	0.182
rs2069840	IL6	G/C	1.72	1.09-2.71	0.019	1.16	0.77-1.76	0.470	1.49	1.01-2.21	0.045	1.24	0.76-2.02	0.396

OR: odds ratio; 95% CI: 95% confidence interval. *p* < 0.05 indicates statistical significance.

## DISCUSSION

Disc degeneration is a process that begins early in life and is a result of various genetic and environmental factors and normal aging [19]. Pathophysiologically, various inflammatory factors play a role in inducing lumbar disc degeneration and nervous radical pain [20]. Although, how environmental and genetic factors modify risk of lumbar disc herniation or gene expression remain uncertain. In this study, we analyzed and associated SNPs of IL4 and IL6 with LDD risk. In summary, four variations (rs1800796, rs1524107, rs2069840, rs2243250) of the selected candidate SNPs were associated with susceptibility to LDD in our study.

IL-6 is a 184 amino acid glycoprotein, an important proinflammatory cytokine produced by activated inflammatory cells, including lymphocytes and macrophages [21]. As an important inflammatory mediator, IL6 plays an important role in rheumatoid arthritis and osteoarthritis. It is considered to be an important mediator of joint destruction and inflammation. [20]. IL6 may function by inhibiting the enzyme that affects the matrix degrading enzyme of the intervertebral disc. Matrix degrading enzyme can change of intervertebral disc matrix proteoglycan, collagen and elastin of biological macromolecules such as structure, function, level and type, weaken the protective effect of fiber ring, the nucleus protruding from the fiber ring is weak [22, 23]. Burke et al. [16]showed that herniated intervertebral disc cells were able to secrete a number of proinflammatory mediators and cytokines, including IL-6, which are accompanied by pain in the lumbar region. There are many IL6 polymorphisms that have been associated with disc degeneration, specifically rs1800797, rs1800796, rs1800795 and rs13006435 [21, 24]. In this study, we found an association of IL-6 SNPs (rs1800796, rs1524107, rs2069840) with an increased risk of developing lumbar disc herniation. Genetic variants in the IL-6 promoter region may lead to aberrant cell transcription and expression. Thereby affecting individual susceptibility to various diseases

In this study, for the first time we focused on anti-inflammatory cytokine, IL-4 in patients with LDD to examine the hypothesis that say LDD could be caused by change in immune system. Studies have shown that the IL-4 polymorphism may affect the function of monocytes, not only producing IL-4, but also producing other cytokines [25]. Remarkably, our results for the first time provided evidence that enhance our understanding of how migraine may relate to, an anti-inflammatory cytokine, IL-4 gene variation. These polymorphisms may lead to changing in IL-4 affinity to their cell targets and consequently unbalance. IL-4 appears to be a prospective target for future development of LDD preventive therapies.

The data presented here must be viewed with caution, because the number of patients is relatively small, so these results should be considered preliminary, this study did not have the ability to convert these SNPs and clinical data, such as pain level, although our data show that IL-4, IL-6 gene polymorphism may be the genetic risk factors for developing LDD in this Han Chinese population.

The results of this study have the guiding significance in clinical work in the future in the treatment of lumbar disc herniation patients, not one-sided that the symptoms of low back pain only from mechanical oppression, inflammatory cytokine stimulation is also very important, can formulate treatment plan to take full account of this, whether the operation should be integrated consider, after surgery treatment for thinking of drug use inhibition of inflammatory factor secretion, whether the long-term follow-up of patients can be detected in serum IL - 4, IL - 6 levels. Whether the author can be refined to the cell level in health examination, whether early screening protrusion of intervertebral disc, in order to achieve the purpose of prevention and treatment of secondary, these are worthy of consideration.

## MATERIALS AND METHODS

Genomic DNA was extracted from 2ml of whole blood which had been obtained from 961 volunteers with their informed consent. Four hundred and ninety-eight patients were recruited from the Second Affiliated Hospital of Inner Mongolia Medical University and The Hohhot First Hospital between 2015 and 2017. All participants had a magnetic resonance imaging (MRI) scan. Data regarding individual characteristics were collected by self-administered questionnaires. Primary exclusion criteria included synovial cysts, spondylolisthesis, spinal tumor, spondylosis, trauma and inflammatory disease. Individuals who had known environmental risk factors, including heavy physical loading, occupational driving, cigarette smoking or obesity (body mass index > 25 kg/m^2^), were also excluded. The control sample consisted of with no history of back problems and with negative MRI findings.

We have selected the ten SNPs described in this study by minor allele frequency (MAF) of > 5% in Chinese Han population and each had an r^2^ of > 0.80 and the SNPs located at IL4 and IL6. Whole blood were used the GoldMag-Mini Whole Blood

Genomic DNA Purification Kit (GoldMag Co. Ltd. Xi’an City, China) extracted. We used a NanoDrop 2000 (Gene Company Limited) were measured DNA concentrations. Sequenom MassARRAY Assay Design 3.0 Software was used to design a Multiplexed SNP MassEXTEND assay [26]. Sequenom MassARRAY RS1000 was used for genotyping, and the related data were managed using Sequenom Typer 4.0 Software [26, 27].

Microsoft Excel and SPSS 20.0 statistical package (SPSS, Chicago, IL) were used to perform statistical analyses. Continuous data were expressed as mean ± SD. Chi-square analysis was used to assess differences in genotype frequencies between cases and controls. Odds ratios (OR), as estimators of relative risk, and 95% confidence intervals (95% CI) were computed using unconditional logistic regression. Associations between the selected SNPs and the risk of LDD were assessed using genotypic model analysis (co-dominant, dominant, recessive, and log-additive) by SNP stats. We used the Haploview software package (version 4.2) and SHEsis software platform for analyses of linkage disequilibrium, haplotype construction, and genetic association. P < 0.05 was considered statistically significant.

## Abbreviations

LDD: lumbar disc disease
SNP: single nucleotide polymorphism
OR: odds ratio
CI: confidence interval
MAF: minor allele frequency
HWE: Hardy-Weinberg equilibrium
